# Report of a Case of Ruptured Spleen

**Published:** 1914-09

**Authors:** E. W. Mulligan, C. W. Hennington

**Affiliations:** Rochester, N. Y.; Rochester, N. Y.


### Report of a Case of Ruptured Spleen

By DR. E. W. MULLIGAN AND DR. C. W. HENNINGTON.

The patient is a robust Italian laborer, aged 32, with no history of illness whatever. On the afternoon of April 16, 1914, he fell backward off a scaffolding, a distance of 15 to 20 feet. His fall was interrupted by striking against a projecting timber. He does not know how he struck the ground. He was not unconscious and says that he was able to rise to his feet, but was brought to the Rochester General Hospital in an ambulance.

He complained of pain across the upper abdomen and a heavy feeling "like a stone in the stomach." He was "afraid of the stomach." In addition, there was great pain in the left shoulder and left hip. His nose was bleeding. A few superficial abrasions of the forehead and face were dressed. He received Morphia gr. 1/8 and was put to bed for observation.

His pulse during the next hours ranged from 80 to 88 and finally to 108. It was not of good quality, being weak and irregular. Temperature 99.8 and respirations 24. He vomited but once, about three cupfulls of dark blood with bright red streaks. He had to be catheterized and 20 ounces of clear urine was obtained which showed the following: 1024 specific gravity, acid, no sugar, a trace of albumen, hyaline and granular casts, no pus and only a rare red blood cell.

From notes of a general physical examination by the house officer, the following points seem of much significance. Both recti muscles somewhat rigid.* Abdomen quite tender, especially in region of spleen, and dull on percussion at outer portion of left upper quadrant and portion of lower quadrant; otherwise abdomen is tympanitic but not distended. Liver, spleen and kidneys not palpable. Liver dullness from 6th rib to the free border. Left hip painful with considerable limitation of motion.

On the following morning the pulse was 100, temperature 99 and respirations 20. He was taking tea and later milk without nausea, and no nausea whatever occurred at any time subsequently. His pulse, however, was not good and he continued to complain of some abdominal pain.

An X-Ray examination was made by Dr. M. B. Palmer with the following report: "No fracture of shoulder. Possible crack of left ilium. Will make another examination."

That afternoon his abdomen had become distended. There was rigidity throughout and of about equal degree. Movable dullness in both flanks. Obliteration of the liver dullness with high tympany. Generalized tenderness. No masses to be felt. The bladder extends more than half way to umbilicus. There is no bruising of the skin. The left iliac crest is painful and the movements of the left hip are restricted. Pressure with both hands over iliac crests causes great pain. Otherwise no changes in the various measurements and examinations. On rectal examination with finger palpating the pelvic brim, there was pain referred to a small area on the inner surface of the left thigh. Haemoglobin 70%, w. b. c. 13,900. Catheterized urine clear.

Operation, April 17, 6 p. m. Extensive right rectus incision from costal border to a point below the umbilicus. Great quantity of dark blood. Evidently no other fluid or exudate. No odor. Peritoneal surfaces smooth and glistening everywhere. Large amounts of blood continued to flood the field. Examination of liver revealed nothing. Palpation of left abdomen in region of spleen and kidney at first revealed nothing. On lifting the transverse colon to examine the retroperitoneum, a large, bruised, dark red, swollen area was seen extending from the midline toward the left under cover of the transverse colon and downward toward the left kidney. The incision was then enlarged by deliberately cutting at right angles across the midline and the left rectus muscle. This revealed the spleen to inspection and showed an organ of normal size with two large tears. The spleen was held firmly suspended from the diaphragm. There was no injury at the hilum. One tear was on the upper surface, probably 6 to 8 cm. in length with the edges spread nearly a cm. The other was a tear extending across the edge of the spleen somewhat lower than the first. Both were sutured with thick catgut, double in the first stitch of each tear and single in the others, a curved blunt needle being used. Some six to eight sutures were taken. The capsule was sufficiently dense not to tear easily. A gauze tampon was inserted and a careful suture of the left rectus fascia and muscle was done, with a hurried closure of the right rectus incision.

Post-Operative History: Intravenous Saline was given and stimulants and subsequently large quantities of water by Murphy method. Pulse was 176 to 140 during the night, and 128 next morning. On the following day 114 with Hb. 60%. On the second day after operation, the pulse was 114 but temperature rose to 103. Patient complained of little except a cough and expectorated a white mucus. Hb. now 45%. W. B. C. 7,400, being Polymorphonuclears 90%, and mononuclears 10%. No plasmodia. The temperature fell gradually. Tampon was started early but not removed completely until the 10th day. Thereafter the temperature stayed normal with a pulse of 70 to 86. Drain closed completely and remainder of wound healed per primam. Due to a misunderstanding, patient was up on the 15th day but ordered back to bed. On the 21st day the blood count was as follows: R. B. C. 3,000,000. Hb. 55%. Another blood count on the 30th day was as follows: R. B. C., 3,260,000. Hb. 60%. A final count on the 42nd day showed: R. B. C. 3,336,000. Hb. 65%, Patient was then allowed to be up and discharged a few days later.

Another X-Ray taken by Dr. Palmer subsequently on May 11th had showed a V-shaped fracture of the ilium, without displacement.

This case of ruptured spleen, therefore, is reported not so much because of the rarity of the condition, nor because of the complicating and misleading fracture of the ilium, but because of the treatment adopted. At present there is an open discussion as to whether we should do a splenectomy or a suture or merely use a tampon. According to the conclusion of Allan F. Barnes, Annals of Surgery, April, 1914, the tampon should be favored as the more physiological method. In our case the suture was required in addition to the tampon. Splenectomy would still be indicated in cases in which the splenic pulp is badly crushed, or in which there is an injury to the vessels at the hilum.

Rochester, N. Y.

---

**Facial Actinomycosis,** following tracheo-bronchial adenopathy and pulmonary generalization. Queyrat and Joltrain, Progres Med., p. 347, July 18, 1914. The diagnosis of mycosis was made by Widal's sero-diagnostic test; radioscopy led to the erroneous diagnosis of aortic aneurysm, the true interpretation of the mass being an enlargement of lmyph nodes, the association of an actinomycotic adenopathy with pulmonary generalization is rare; potassium iodid gives favorable results but is badly borne by the patient, who shows progressive dyspnoea, cough and expectoration.

---

**Retrogressive Virilism.** Dalche, Progres Med., p. 347, July 18, 1914, observed in 1912 a woman with amenorrhoea and a small mass in the left cul de sac, considered to be a slightly enlarged tube. She had a beard and the features were masculine. Recently, she was again seen. Menstruation had been restored though still scanty and the hirsuties has diminished. No obesity. He considers the virilism due to suspension of ovarian function and not due to adrenal lesion.

---

**Xanthochromism Palmo-Plantar,** was illustrated in a case of Labbe and Bith (ibidem) as an undescribed symptom of diabetes. No pigmentation of mucosa nor urinary jaundice.

